# Metastasis of follicular thyroid carcinoma to the maxillary sinus

**DOI:** 10.4103/0972-3919.78254

**Published:** 2010

**Authors:** Arvind Krishnamurthy, Anitha Vaidhyanathan, Kumar R Krishna

**Affiliations:** Department of Surgical Oncology, Cancer Institute (WIA), Adyar, Chennai, India; 1 Department of Nuclear Medicine, Cancer Institute (WIA), Adyar, Chennai, India

**Keywords:** Distant metastasis, paranasal sinuses, thyroid neoplasms

## Abstract

Thyroid carcinoma metastatic to the paranasal sinuses is extremely rare. We report a case of follicular thyroid carcinoma metastatic to the right maxillary sinus, with extension into the right side of the hard palate in a young lady. A radioactive iodine-131 (I-131) scan post total thyroidectomy revealed 0.8% uptake in the neck; the whole body scan revealed a functioning metastasis in the region of the right maxillary antrum. Pathological confirmation of metastasis was done by correlating the fine needle aspirate cytology with the thyroidectomy histology. The patient initially received 40 Gy of external beam radiotherapy, subsequently, she received 110 millicuries of I-131, followed by 50 millicuries 6 months later. She continues to be on follow-up on Eltroxin™ suppression and has remained disease free for the past 4 ½ years.

## INTRODUCTION

Follicular carcinoma is the second most common cancer, accounting for 15%–20% of all thyroid gland malignancies. Distant metastasis, although relatively uncommon, has been known to occur to the lungs, bone, brain, and soft tissues. Metastasis to the paranasal sinuses (PNS) is extremely rare and accounts for 1% of all the malignant diseases of the mouth and jaw.[1,2] Most cases of metastasis are from primaries below the clavicle.[2] It is widely accepted that as the jaw does not contain a lymphatic system, metastasis to the jaw occurs via the blood stream.[2] An explanation regarding the etiopathogenesis of PNS metastases was proposed by Nahum and Bailey:[3] they implicated the vertebral venous plexus, a highly communicable system, through which retrograde spread of tiny emboli could occur to the PNS. The other route of spread is via the lymphatics between the thyroid and the skull base and sinuses.

We present a rare case of metastasis of follicular thyroid carcinoma to the maxillary sinus that was treated with external radiation and radioactive iodine.

## CASE REPORT

A 31-year-old female was referred to our center with a histological diagnosis of follicular carcinoma. A month earlier, a near total thyroidectomy had been done for a multinodular goiter. She had no comorbid illnesses, and on clinical examination the thyroid bed and neck were found to be normal. We noticed fullness in the region of the right maxillary sinus, along with a bulge in the right side of the palate. The patient had in fact noticed the palatal bulge 6 months earlier, but it had been associated with pain only for about a month.

**Figure 1:**
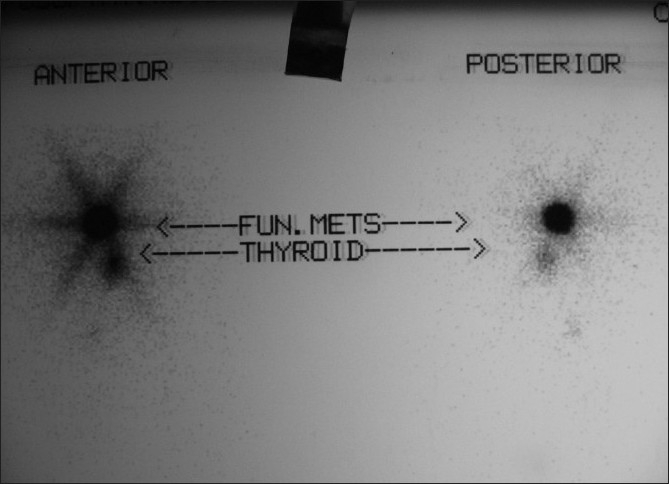
I-131 scan post thyroidectomy prior to first ablation

**Figure 2:**
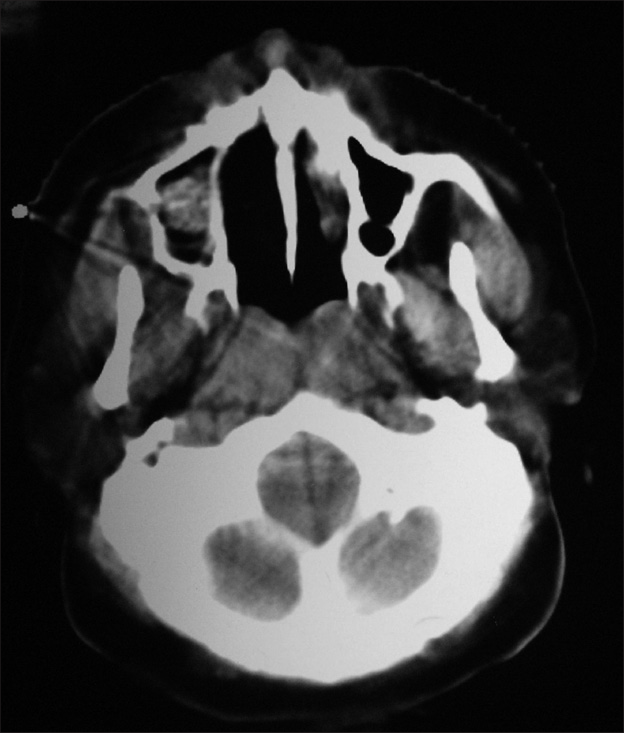
Axial CT scan showing tumor in the right maxillary sinus

**Figure 3:**
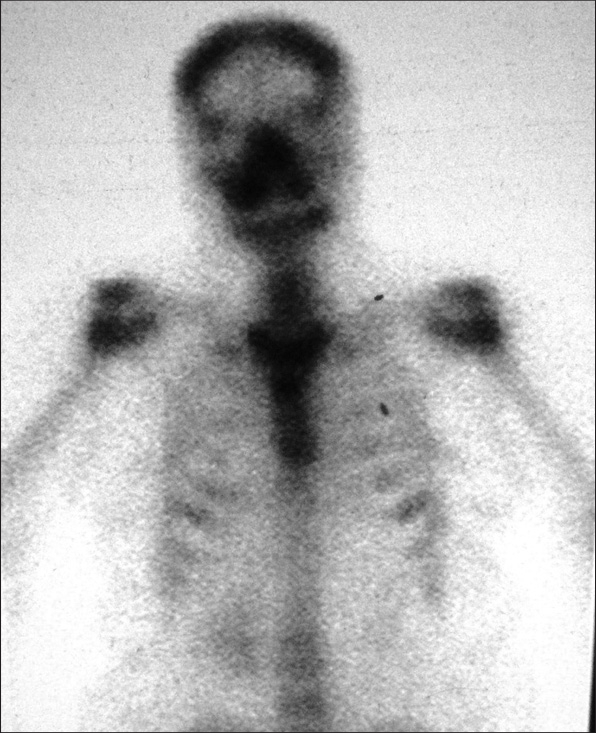
Bone scan showing increased uptake in the right maxillary sinus

**Figure 4:**
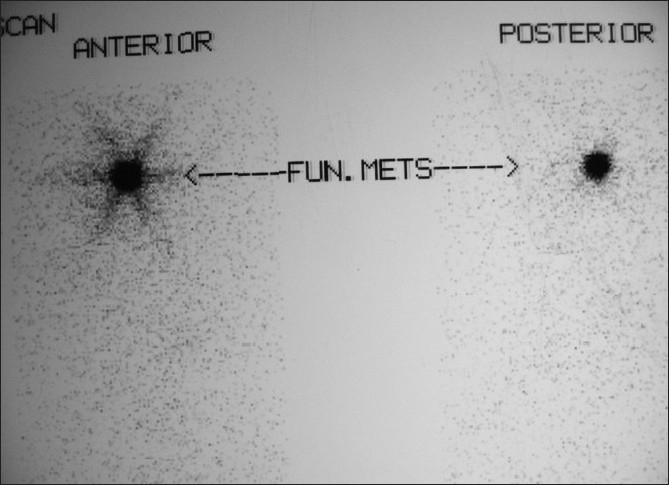
Aspiration cytology from the lesion in the maxillary sinus

A radioactive iodine-131 (I-131) scan revealed 0.8% uptake in the neck; the whole body scan revealed a functioning metastasis in the region of the right maxillary sinus [Figure 1]. A CT scan [Figure 2] of the head and neck and a bone scan [Figure 3] done subsequently confirmed the presence of a 6 × 4 cm mass in the right maxillary sinus. Aspiration cytology from the bulge showed atypical follicular cells, which on correlation with the slides and blocks of the thyroidectomy specimen was suggestive of metastatic involvement from a follicular carcinoma of the thyroid [Figure 4]. The patient was not keen on surgery for the maxillary sinus lesion and was hence offered external beam radiotherapy and radioiodine therapy.

**Figure 5:**
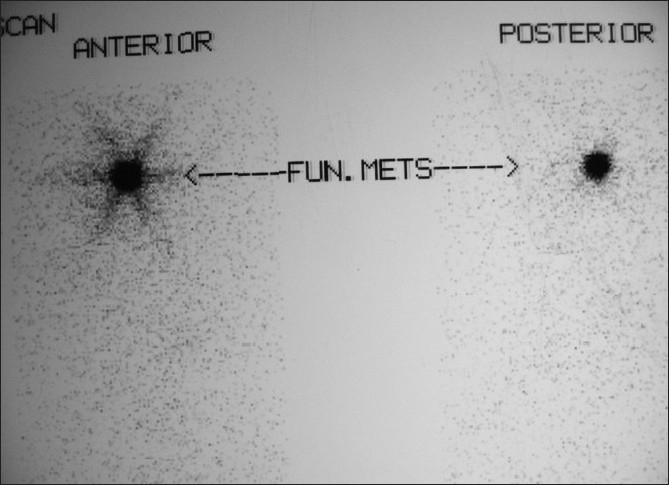
I-131 scan prior to the second ablation

She initially received 40 Gy of Cobalt-60 external beam radiation from 22/10/2005 to 22/11/2005. In view of persistent uptake in the right maxillary sinus [Figure 5], she went on to receive 110 millicuries of I-131 in December 2005 and 50 millicuries of I-131 in June 2006. Her subsequent I-131 scans as well as serum thyroglobulin levels were within normal limits. There was clinical regression of the right maxillary lesion as well. She has been on regular follow-up for close to 4½ years and continues to be disease free on Eltroxin™ suppression.

## DISCUSSION

The great majority of carcinomas affecting the PNS are primary carcinomas, metastatic involvement being relatively rare. Bernstein *et al*.[4] reviewed 82 cases with metastatic foci in the PNS and reported that 40 patients had lesions in the maxilla, 15 in the ethmoids, 12 in the frontal sinus, and 6 in the sphenoid. Other sites included the nose, nasopharynx, palate, and alveolar ridge. In the majority of cases the primary tumor was in the kidney (55%), followed by the bronchus and urogenital ridge (11% each), the breast (10%), the gastrointestinal tract (6%), and the thyroid (3%). The references to thyroid metastases were all from the European literature, with descriptions of metastasis to the sphenoid, skull base, lung, nasal cavity, and frontal sinus. There have been only isolated case reports of thyroid tumors metastatic to the PNS. The commonest histology reported is follicular carcinoma, and a trend towards multifocality of PNS involvement has been noted.[5] A female preponderance has also been reported.

CT scan is generally considered the method of choice for delineating bony involvement and erosions due to metastatic lesions to the PNS; MRI gives better soft tissue resolution. Bone scanning with technetium-99 is considered important for general staging of malignant tumors when there is concern for possible bone metastases.

The treatment of these metastatic tumors requires careful consideration as sphenoid and skull base involvement is common, thus often precluding surgical extirpation of the metastatic lesions.

Radioactive iodine treatment is considered to be the first line of treatment for distant metastasis from thyroid carcinomas that concentrate a significant amount of radioiodine. Although a well-differentiated follicular thyroid carcinoma very often concentrates radioiodine quite rapidly and responds well to I-131 therapy, a lesser number of metastatic lesions have the ability to concentrate radioactive iodine to achieve successful treatment as seen in our patient.

Unlike patients with regional metastasis, for whom surgery is the primary treatment modality, the indications for surgery are few when it comes to distant metastasis. Surgical removal of the resectable lesions, with or without external beam radiation therapy, should be considered for these patients, especially those that are not concentrating iodine.

Distant metastasis is the principal cause of death in cases of well-differentiated thyroid carcinomas. About 10% of papillary carcinomas and 25% of follicular carcinomas develop distant metastasis, with about 50% of patients having such metastasis at the time of diagnosis. The prognosis of these patients is poor, and over 50% of patients are likely to die within 5 years, irrespective of the histology of the tumor.[6] Some patients, especially the younger ones, survive longer. Our patient has been disease free for close to 4½ years now.

It is the authors’ opinion that while examining patients with a previous history of a thyroidectomy, the head and neck surgeon should maintain a high index of suspicion regarding the possibility of metastatic spread. Although metastasis to the PNS is rare, pertinent symptoms such as facial swelling or bulge, epistaxis, nasal obstruction, visual disturbances, and cranial nerve abnormalities should be rigorously investigated and treated.
